# Peripheral Immune Cell Infiltration in the Hippocampus of Sepsis Mice

**DOI:** 10.1111/jcmm.70872

**Published:** 2025-10-31

**Authors:** Hui Zhang, Meixian Zhang, Yanan Gao, Qian Chen, Qiang Liu, Fanbing Meng, Xiaoxiao Sun, Miaomiao Fei, Cheng Li, Lize Xiong

**Affiliations:** ^1^ Department of Anesthesiology and Perioperative Medicine, Shanghai Key Laboratory of Anesthesiology and Brain Functional Modulation, Clinical Research Center for Anesthesiology and Perioperative Medicine, Translational Research Institute of Brain and Brain‐Like Intelligence, Shanghai Fourth People's Hospital, School of Medicine Tongji University Shanghai China

**Keywords:** hippocampus, immune cell, infiltration, neuroinflammation, sepsis‐associated encephalopathy

## Abstract

Peripheral immune cell infiltration plays a key role in the excessive intracranial inflammatory response during sepsis, contributing to cognitive dysfunction and increased mortality. This study aims to elucidate the immune inflammatory status of hippocampal tissue in septic mice, providing potential targets for treatment and prevention. Male C57BL/6J mice underwent cecal ligation and puncture (CLP) or sham surgery. Twenty‐four hours post‐surgery, hippocampal tissues were harvested for transcriptome sequencing and immunofluorescence analysis. Immune cell infiltration was assessed using the xCell package, and structural changes in brain microvessels were evaluated by transmission electron microscopy. Flow cytometry and immunofluorescence confirmed immune cell presence in the hippocampus. Hub genes related to immune responses were identified using protein–protein interaction networks, weighted gene co‐expression network analysis, and enrichment analysis of immune‐related biological processes. In CLP mice, microvessel endothelial cells were separated, with swelling and damage to tight junctions. Significant infiltration of myeloid immune cells, including dendritic cells, monocytes and neutrophils, was observed in the hippocampus. Seven immune‐related hub genes (*Fcer1g*, *Fcgr2b*, *Fcgr3*, *Icam1*, *Itgb2*, *Ptprc* and *Slc11a1*) play a pivotal role in mediating immune responses. *Fcer1g*, *Fcgr2b* and *Slc11a1* were positively correlated with monocytes and neutrophils, suggesting their involvement in the recruitment of immune cells to the brain. This study enhances our understanding of peripheral immune cell infiltration in sepsis and provides novel insights into potential therapeutic targets for sepsis‐associated encephalopathy.

## 1 Introduction

Sepsis‐associated encephalopathy (SAE) is characterised by widespread cerebral dysfunction and arises as a consequence of a systemic inflammatory response to infection, rather than from a direct infection of the central nervous system [1]. It causes delirium, entry into a coma state, and cognitive impairment. Approximately 20%–50% of sepsis patients experience delirium [2], and 70% of SAE patients exhibit neurological symptoms, ranging from lethargy to coma [3], with over 80% of patients showing electroencephalogram abnormalities [4]. Furthermore, SAE patients have an elevated mortality rate and a 2.22‐fold increased risk of long‐term dementia compared to sepsis patients without SAE [5, 6]. Patients experiencing acute mental status alterations due to encephalopathy exhibit a nearly 49% higher mortality rate than those with pre‐existing mental status changes (41%) or those maintaining normal mental status (26%) [7], suggesting that the central nervous system status significantly influences mortality. SAE is typically caused by excessive brain inflammation, which is closely related to neurological disturbances [1, 8].

In sepsis, the infiltration of peripheral immune cells is a crucial step in the excessive activation of intracranial inflammation. The immune system releases inflammatory mediators such as cytokines and chemokines, which attract leukocytes in response to the intracranial infection. These immune cells infiltrate the brain parenchyma due to increased permeability, upregulation of leukocyte adhesion molecules and compromise of the blood–brain barrier (BBB) integrity [9, 10]. While this immune response can help combat infection, excessive inflammation and immune cell infiltration can damage brain tissue. Inflammatory mediators secreted by immune cells initiate an inflammatory response in the brain, leading to neuroinflammation and neuronal damage [11, 12]. When monocytes infiltrate the brain, they typically differentiate into macrophages or resident microglia. These cells release a range of pro‐inflammatory cytokines, chemokines and oxidative molecules, such as nitric oxide (NO) and reactive oxygen species (ROS), which contribute to the induction of neuroinflammation. This inflammatory response can result in neuronal damage and degeneration. Furthermore, the secretion of pro‐inflammatory mediators disrupts the BBB, thereby facilitating the infiltration of additional immune cells and inflammatory agents, which exacerbates neuronal injury and further compromises the integrity of the central nervous system. Additionally, immune cells induce oxidative stress and the production of free radicals within the brain, further harming neurons and surrounding cells [9].

The pivotal role of peripheral immune cell infiltration in the progression of SAE indicates that it is crucial to develop strategies to minimise the harmful effect of peripheral immune cells on the central nervous system, thereby improving patient outcomes. However, the specific types of peripheral immune cell infiltration in the brain remain unclear. Clarifying the types of peripheral immune infiltration will help provide clues for treatment. This study aims to characterise the immunoinflammatory status of hippocampal tissue in sepsis mice at the transcriptional level. Our analyses revealed that monocytes, neutrophils and dendritic cells infiltrate the central nervous system during sepsis, providing valuable insights into the pathogenesis of SAE.

## 2 Materials and Methods

### 2.1 Animal Model

The study was approved by the Local Ethics Committee of Tongji University (ethical committee number: TJBH07024104). Male C57BL/6J mice, aged 8–10 weeks, were purchased from Shanghai Jihui Laboratory Animal Care Co. Ltd. (Shanghai, China). The animals were housed in the Tongji University Laboratory Animal Center, maintained under a 12‐h light/dark cycle, and provided with ad libitum access to food and water. A 7‐day acclimatisation period was allowed before the experiments began. The mice were then randomly assigned to undergo either a sham operation or cecal ligation and puncture (CLP), a procedure known to induce sepsis [13]. Anaesthesia was induced with 2% sevoflurane, followed by an abdominal incision. The cecum was ligated approximately 1.0 cm from its distal end and subsequently punctured using an 18‐gauge needle. Before repositioning the cecum into the peritoneal cavity, a small amount of faeces was gently expressed to confirm the patency of the puncture site. Sham operation mice only underwent laparotomy without CLP. Following layered closure of the abdominal incision, 50 mL/kg of prewarmed normal saline was administered subcutaneously, and 3 mg/kg bupivacaine and 0.1 mg/kg morphine were injected subcutaneously around the incision to manage postoperative pain.

### 2.2 Hippocampal Tissue Separation

Twenty‐four hours post‐surgery, no significant change in body weight was observed in the mice. The hippocampus was carefully extracted. The mice were removed from their cages, decapitated at the foramen magnum immediately upon removal. Any blood on the skull's surface was rinsed with saline at 0°C–4°C. The skin was then incised to reveal the skull, which was meticulously opened along the midline using surgical scissors, ensuring minimal disturbance to the brain tissue. Once the brain was fully exposed, the meninges and surface blood vessels were delicately removed using ophthalmic forceps. The brain was lifted from the base of the skull, briefly immersed in saline at 0°C–4°C for 30 s, and placed on an ice pack to facilitate hippocampal isolation. After isolation, the hippocampus was quickly snap‐frozen in liquid nitrogen, sealed, and stored at −80°C for preservation.

### 2.3 mRNA Sequencing

In accordance with the established protocol, total RNA was extracted using the TRIzol reagent kit (Invitrogen, Carlsbad, CA, USA). The integrity and quality of the RNA were assessed by RNase‐free agarose gel electrophoresis. Eukaryotic mRNA was subsequently enriched using oligo(dT) beads and fragmented with a fragmentation buffer to facilitate reverse transcription into complementary DNA (cDNA), resulting in double‐stranded cDNA fragments. These cDNA fragments underwent end repair, addition of an adenine base at the 3′ ends (A‐tailing), and ligation with Illumina sequencing adapters. The prepared cDNA library was then amplified by polymerase chain reaction (PCR) and sequenced using the Illumina platform.

### 2.4 Transcriptome Sequencing and Data Analysis

Raw reads from sequencing may include adapters or low‐quality bases, which can compromise compromising subsequent assembly and analysis. To generate high‐quality reads, additional filtering was performed with fastp (version 0.18.0) [14]. Reads that contained adapters, exhibited more than 10% unknown nucleotides, or had over 50% low‐quality bases were excluded from further analysis. Subsequently, the Bowtie2 tool (version 2.2.8) was employed to align the reads to the ribosomal RNA (rRNA) database, allowing for the removal of reads mapping to rRNA [15]. The remaining high‐quality reads were utilised for gene assembly and the quantification of gene abundance. Paired‐end reads were aligned to the reference genome with HISAT2 [16], and StringTie (v1.3.1) was used to assemble the aligned reads for each sample [17, 18]. Gene expression levels and transcript region variability were quantified using RSEM software [18], which normalises for gene length variations and sequencing depth, enabling the direct comparison of gene expression across samples. DESeq2 software was used for detecting differential expressed RNAs between different groups [19]. Differentially expressed genes (DEGs) were classified with an absolute fold change greater than 1 and adjusted *p* values less than 0.05.

### 2.5 Functional Enrichment of DEGs

Functional enrichment of DEGs following CLP surgery was performed using Metascape (http://metascape.org) for gene function annotation and prioritisation [20, 21]. Gene ontology (GO) biological processes and Kyoto Encyclopedia of Genes and Genomes (KEGG) enriched in the DEGs were analysed to gain insights into the underlying biological changes associated with sepsis [22].

### 2.6 Peripheral Immune Cell Infiltration

The xCell software package was used to evaluate differences in hippocampal immune cell infiltration between the CLP and control groups. This evaluation was conducted by calculating scores for various immune and stromal cell types, utilising a gene signature approach that encompasses 10,808 genes [21, 23]. Of the 34 immune cell types examined, 12 were myeloid cells. Before analysis, mouse genes were converted to their human homologues using Biomart [24], because xCell assesses immune infiltration based on human gene sets.

### 2.7 Immune‐Related Hub Genes Screening

To investigate the genes closely associated with the changes in immune‐related biological processes in the hippocampus of mice after CLP, we adopted an integrated approach utilising three hub gene screening methods. Initially, hub genes were identified from the protein–protein interaction (PPI) network of DEGs from the STRING database by employing 12 different algorithms in the cytoHubba application [25, 26]. Secondly, hub genes were identified through the application of weighted gene co‐expression network analysis (WGCNA). This method detects highly correlated gene modules and ascertains the hub genes within these modules [27]. The specific steps involved in this process were as follows: (1) the soft threshold parameter β was set to 9, signifying strong gene correlations, with a threshold value of 0.8, to construct a scale‐free network; (2) the dynamic tree‐cutting algorithm was employed to identify gene modules, resulting in 20 distinct modules following the merger of highly correlated coefficients; (3) we calculated correlations between sample group phenotypes and modules with correlation coefficients > 0.6 and *p* values < 0.05; (4) hub genes within the selected modules were screened based on criteria such as an absolute gene significance (GS) value exceeding 0.8, module membership (MM) > 0.8 and a *q*‐weighted value < 0.05. Thirdly, we identified hub genes involved in immune‐related biological processes enriched in DEGs. The intersection of PPI hub genes, module hub genes and immune‐related biological process hub genes was considered a set of hub genes.

### 2.8 Western Blot

Tissues were lysed with protein lysis buffer (Thermo Fisher Scientific, USA) with protease inhibitors (Bimake, China). Protein concentrations were quantified using a Pierce BCA protein assay kit (Beyotime, China). Based on the protein concentration results, 10 μg of total protein was used for detection. Whole proteins were transferred onto PVDF membranes (Bio‐Rad, USA). The primary antibodies included a β‐actin antibody (1:1000 dilution, #4967, Cell Signalling Technology, USA), a caspase‐3 antibody (1:1000 dilution, #9662, Cell Signalling Technology) and a cleaved caspase‐3 (C‐caspase‐3) antibody (1:1000 dilution, #9664, Cell Signalling Technology). The secondary antibodies were a rabbit IgG for horseradish peroxidase (HRP)‐linked antibody and a rat IgG for HRP‐linked antibody purchased from Cell Signalling Technology. The bands were visualised using western chemiluminescence (Clarity Western ECL Substrate, Bio‐Rad) and then quantified using a ChemiDoc XRS + system (Bio‐Rad). β‐Actin was used to normalise the target protein levels and control for loading differences in the total protein amount.

### 2.9 ELISA

We measured the proinflammatory cytokines in hippocampal tissue using the IL‐1b, IL‐6 and TNF‐Mouse ELISA Kits (Elabscience, China), following the manufacturer's instructions.

### 2.10 Immunofluorescence and Immunohistochemical

Immunofluorescence analysis was conducted to assess peripheral immune cell infiltration in the mouse hippocampus. The hippocampal tissues were fixed in 4% paraformaldehyde for a duration of 4 h at a temperature of 4°C, then immersed overnight in a 30% (w/v) sucrose solution prepared in 0.1 M phosphate buffer, also maintained at 4°C. Finally, the brains were sectioned into 30 μm thick slices utilising a freezing microtome. Sections were incubated overnight at 4°C with primary antibodies against CD11c (1:100 dilution, Cell Signalling Technology, Danvers, MA), Ly6c (1:100 dilution, Abcam, Cambridge, UK), or ZO‐1 (1:100 dilution, Abcam, Cambridge, UK). After thorough washing, the sections were incubated with the corresponding secondary antibodies at room temperature for 50 min. Nuclei were counterstained with 4′,6‐diamidino‐2‐phenylindole (DAPI) (Wuhan Servicebio Technology Co. Ltd., Wuhan, China) for 10 min at room temperature. Images were acquired utilising an FV3000 confocal microscope (Olympus, Tokyo, Japan).

Immunohistochemical analysis was conducted to assess the expression of specific proteins in the hippocampus. Mouse hippocampal tissue sections were dewaxed and rehydrated, followed by antigen retrieval by boiling in sodium citrate buffer (pH 6.0). After cooling to room temperature, endogenous peroxidase activity was blocked by incubation in 3% hydrogen peroxide for 30 min in the dark. The tissue was then blocked with 3% BSA. Sections were incubated overnight at 4°C with the primary antibodies. After three washes with PBS, the tissue was incubated with the appropriate secondary antibody for 50 min at room temperature. Following slight drying, freshly prepared DAB solution was applied to visualise the target proteins. Haematoxylin was used for nuclear counterstaining, and sections were examined using an XSP‐C204 microscope. The antibodies used in this study are listed as follows: CD45 (Ptprc, 1:400 dilution, Proteintech, 20103‐1‐AP), ICAM1 (1:100 dilution, AiFang Biological, AF0688), CD18 (Itgb2, 1:100 dilution, Affinity Biosciences, DF6896), NRAMP1 (Slc11a1, 1:200 dilution, BOSTER, A02547‐3), CD32 (Fcgr2b, 1:200 dilution, Affinity Biosciences, AF04227), CD64 (Fcgr1g, 1:100 dilution, Affinity Biosciences, AF13220).

### 2.11 Transmission Electron Microscopy

The hippocampal tissue was dissected into small pieces (approximately 1–2 mm^3^) and fixed with 2.5% glutaraldehyde at 4°C. Subsequently, the tissue was fixed with 1% osmium tetroxide in 0.1 M phosphate buffer (pH 7.4) at room temperature for 2 h. The tissue was then dehydrated through a graded ethanol series (30%, 50%, 70%, 80%, 85%, 90% and 100%). Following dehydration, the tissue was infiltrated with a mixture of acetone and epoxy resin (2:1), and then with acetone and epoxy resin (1:1). Epoxy resin infiltration was performed in a 37°C incubator. The infiltrated tissue was placed in an embedding mould, and fresh epoxy resin was added. After polymerisation at 60°C for 48 h, 80–100 nm thin sections were prepared. Following uranyl‐lead double staining, the sections were observed under a transmission electron microscope (FEI Company, TECNAI G 20 TWIN).

### 2.12 Flow Cytometry

Immune cells in the brain were analysed by flow cytometry. Hippocampal tissues were dissected and immediately transferred to ice‐cold PBS. After gentle mincing, trypsin was added for digestion and incubated at 37°C for 20 min with gentle shaking. Digestion was terminated by adding FBS, and the resulting cell suspension was filtered through a 70 μm mesh to remove clumps. Myelin‐containing supernatant was discarded by centrifugation at 3000 rpm for 5 min at 4°C. The cells were resuspended in RPMI 1640 medium, and 500 μL of the cell suspension (containing 2 × 10^6^ cells) was transferred to a flow cytometry tube. The cells were then incubated with Human TruStain FcX (BioLegend, 422301) at room temperature for 10 min to block non‐specific binding. After centrifugation, the supernatant was discarded. The following antibodies were used to stain macrophage subsets, monocytes, neutrophils and dendritic cells in the brain: mouse anti‐F4/80 PE (1:100, BioLegend, 123110), mouse anti‐CD206 APC (1:100, BioLegend, 141708), mouse anti‐CD86 FITC (1:100, BioLegend, 105110), mouse anti‐CD11b FITC (1:100, BioLegend, 101205), mouse anti‐Ly6C PE (1:100, Elabscience, E‐AB‐F11200), mouse anti‐Ly6G PE (1:100, Elabscience, E‐AB‐F11200), mouse anti‐CD11c FITC (1:100, BioLegend, 117305) and mouse anti‐MHC II PE (1:100, Elabscience, E‐AB‐F0990D). Data were acquired using an ACEA NovoCyte flow cytometer (Agilent) and analysed with FlowJo software.

### 2.13 Statistical Analysis

Transcriptomic data were analysed and visualised using the appropriate R packages within R software version 4.2.2. Data transformation and analysis were conducted with packages such as dplyr, reshape2 and tidyverse, while visualisation was achieved using ggplot2, ggpubr, ggstatsplot, pheatmap, RColorBrewer and VennDiagram. The Mann–Whitney *U* test was used to assess differences in cell types between groups, and Pearson correlation analysis was applied to examine associations between immune cells and genes. Flow cytometry results were expressed as mean ± SD, with comparisons between groups made using the Student's *t*‐test. A *p*‐value of less than 0.05 was considered statistically significant.

## 3 Results

### 3.1 Immunological Biological Processes Were Activated in the Hippocampus Following of Mice After CLP

**FIGURE 1:**
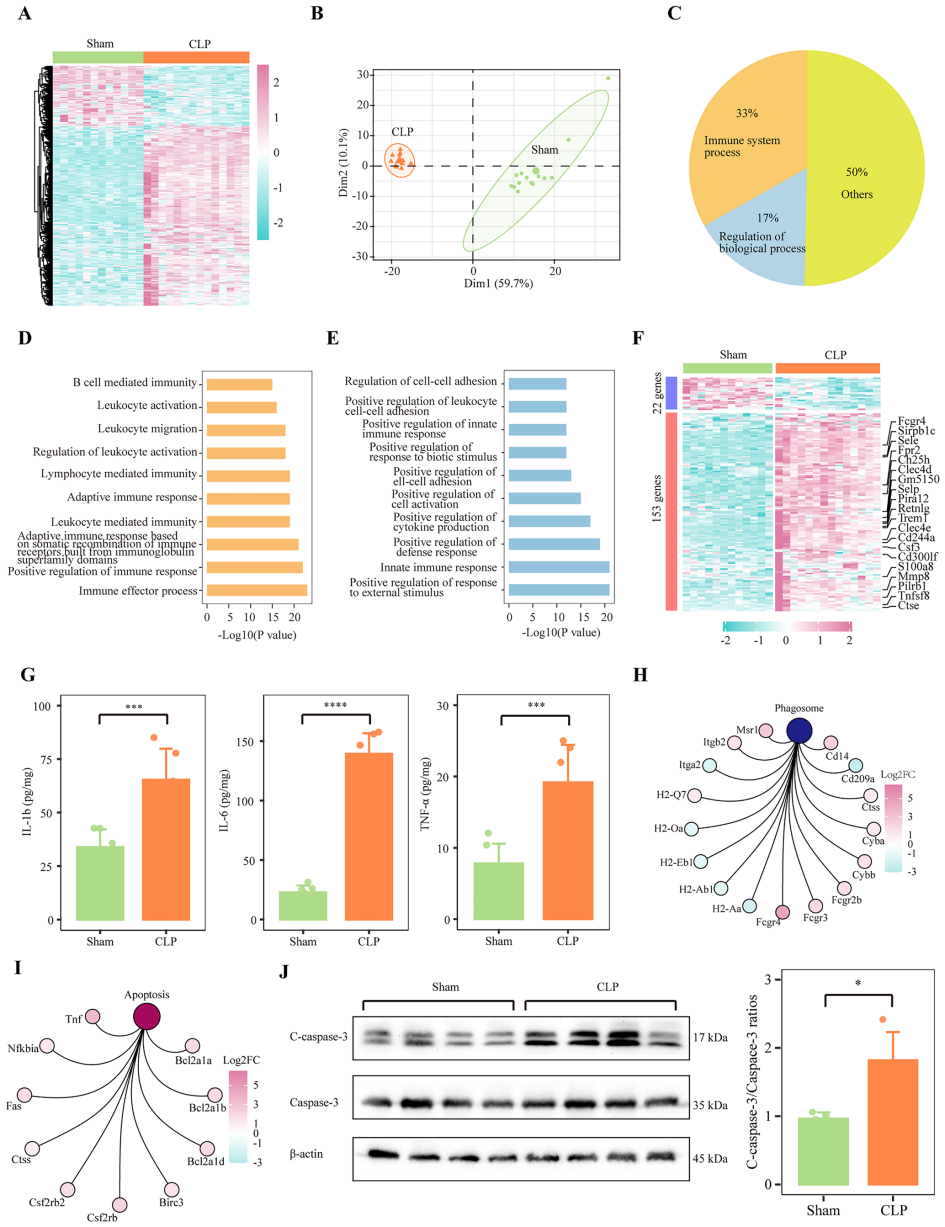
Active immune status in the hippocampus after CLP. (A) Heatmap of 504 differentially expressed genes (DEGs), with 378 upregulated and 126 downregulated in CLP mice compared to the sham group. (B) PCA based on DEGs from all samples. (C) GO enrichment analysis of immune‐related biological processes in DEGs. (D) Top 10 immune‐related biological processes. (E) Top 10 regulatory processes linked to immune functions. (F) Heatmap of 175 immune‐related DEGs, with the top 20 showing consistent upregulation in the CLP group. (G) Level of IL‐1b, IL‐6 and TNF‐α in the hippocampus (***p < 0.001, ****p < 0.0001). (H) Expression heatmap of DEGs enriched in phagosome pathways. (I) Expression heatmap of DEGs enriched in apoptosis pathways. (J) Level of apoptosis in the hippocampus (*p < 0.05). (A–H). DEGs in the gene signatures related to aDC, basophils, eosinophils, monocytes, macrophages M2, mast cells, neutrophils, and pDC. CLP, cecal ligation and puncture; DEG, differentially expressed genes.

A total of 26 hippocampal tissue samples from mice were subjected to transcriptomic sequencing, including 12 samples from the sham group and 14 samples from the CLP group. As the read count increased, the detection of genes approached a saturation threshold, with approximately 75% of the total genes identified once the read count exceeded 3 × 10^8^ (Figure S1). The correlation coefficient between samples exceeded 98%, indicating a high degree of consistency (Figure S2A). Row counts for each sample were normalised using the vst function. The overall gene expression for each sample is shown in Figure S2B. DEGs were identified with an absolute fold change greater than 1 and adjusted *p* values less than 0.05. Compared to the sham group, the CLP group exhibited 378 upregulated genes and 126 downregulated genes. A heatmap of all DEGs is shown in Figure 1A. Principal component analysis (PCA) grouped the samples into two clusters based on DEGs (Figure 1B). GO enrichment analysis revealed that 33% of the enriched biological process terms were related to immune system processes, and 17% were associated with the regulation of biological processes (Figure 1C). The top 10 immune system‐related biological processes included the regulation of leukocyte‐mediated immunity, leukocyte activation and migration, and adaptive immune responses (Figure 1D). The top 10 biological processes related to regulation included the regulation of innate immune responses and leukocyte cell–cell adhesion (Figure 1E). Of the 175 DEGs associated with immune system processes, most were upregulated in the CLP group, with the top 20 showing consistent upregulation (Figure 1F). The potential driver cytokines IL‐1b, IL‐6 and TNF‐α were highly expressed after the surgery (Figure 1G). KEGG enrichment analysis showed that phagosome and apoptosis pathways related to cellular destruction were significantly enriched by DEGs; most enriched genes were highly expressed in CLP (Figures 1H,I and S3). In addition, increased apoptosis was observed in the hippocampal tissue of CLP mice (Figure 1J). Overall, these results suggested that immune‐related biological processes were activated and that significant tissue damage occurred in the hippocampus of mice following CLP.

### 3.2 Myeloid Immune Cell Infiltration Scores Were Significantly Increased After CLP

**FIGURE 2:**
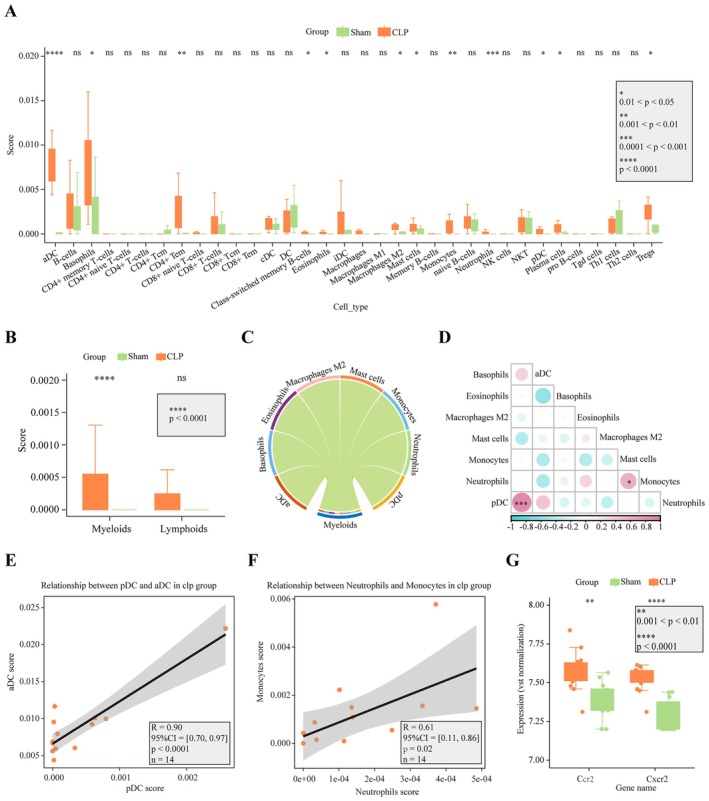
Increased myeloid immune cell infiltration in the hippocampus of CLP mice. (A) Immune infiltration scores for various cell types (median ± IQR). (B) Myeloid cell infiltration was significantly higher in the CLP group compared to the sham group (mean ± SD). (C) Infiltration scores were primarily driven by aDCs, basophils, eosinophils, M2 macrophages, mast cells, monocytes, neutrophils, pDCs and plasma cells. (D, E) Positive correlation between pDCs and aDCs (*R* ^2^: 0.90, 95% CI: 0.70–0.97, *p* < 0.0001). (D, F) Positive correlation between neutrophils and monocytes (*R* ^2^: 0.61, 95% CI: 0.11–0.86, *p* = 0.02). (G) Ccr2 and Cxcr2 were significantly overexpressed in the hippocampus of CLP mice (median ± IQR). aDCs, activated dendritic cells; CI, confidence interval; CLP, cecal ligation and puncture; pDCs, plasmacytoid dendritic cells.

**FIGURE 3:**
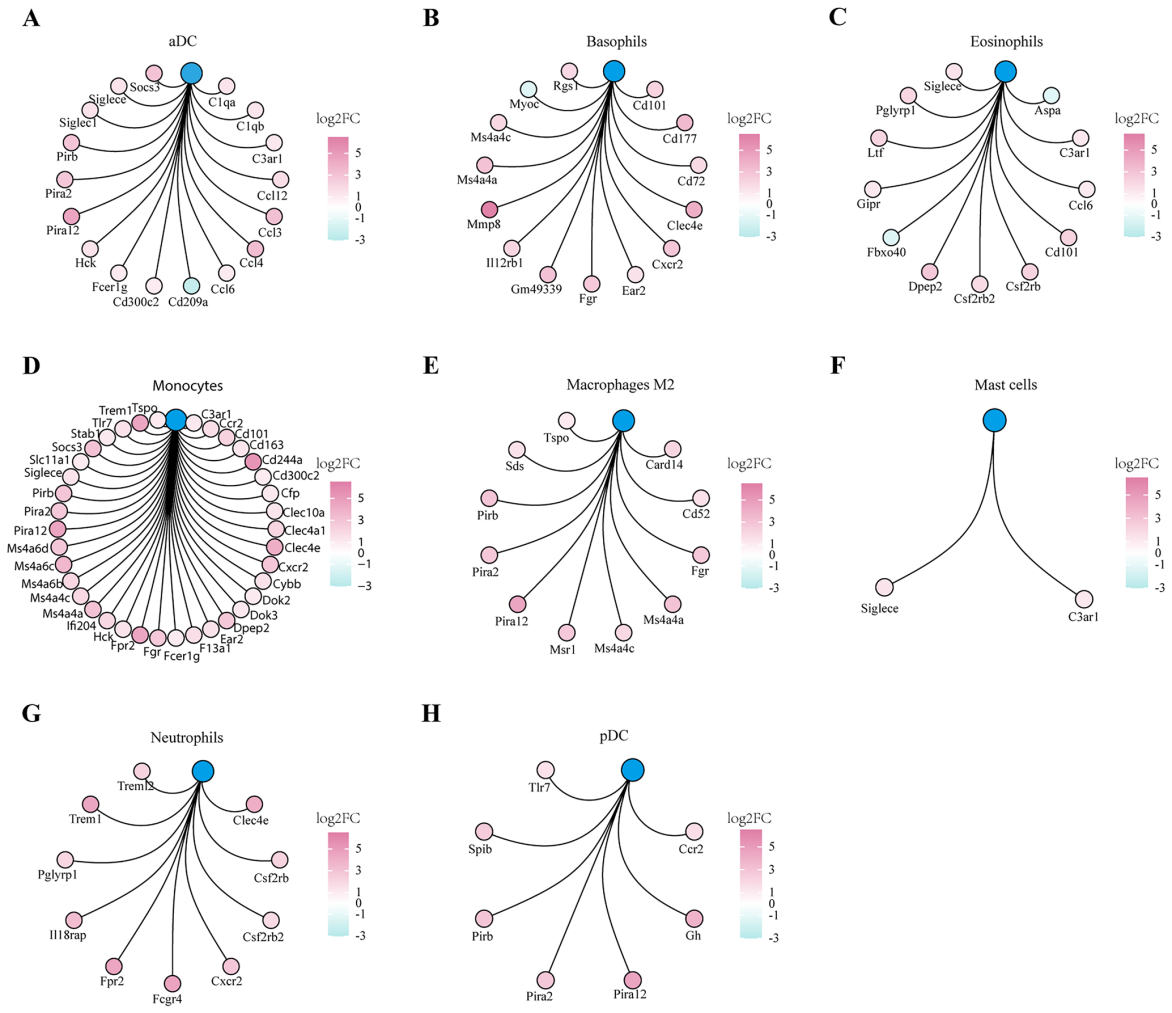
Upregulation of DEGs in myeloid lineage cells in the CLP group. (A–H). DEGs in the gene signatures related to aDC, basophils, eosinophils, monocytes, macrophages M2, mast cells, neutrophils, and pDC CLP, cecal ligation and puncture; DEG, differentially expressed genes.

Immune infiltration scores were calculated using the xCell package after converting approximately 80% of the 22,148 mouse genes to their human homologues. The infiltration scores for 34 immune cell types are presented in Figure 2A. Several cell types, including activated dendritic cells (aDCs), basophils, CD4^+^ T cells, class‐switched memory B cells, eosinophils, M2 macrophages, mast cells, monocytes, neutrophils, plasmacytoid dendritic cells (pDCs), plasma cells and regulatory T cells (Tregs), exhibited higher scores in the CLP group compared to the sham group. Notably, a significantly higher infiltration score for myeloid lineage cells was observed in the CLP group (*p* < 0.05), while no significant difference was found for lymphoid lineage cells (Figure 2B). The difference in myeloid lineage cell scores was primarily attributed to changes in aDCs, basophils, eosinophils, M2 macrophages, mast cells, monocytes, neutrophils, pDCs and plasma cells (Figure 2C). Pearson correlation analysis revealed a positive correlation between pDCs and aDCs (*R* ^2^: 0.90, 95% confidence interval [CI]: 0.70–0.97, *p* < 0.0001, Figure 2D,E) and a positive correlation between neutrophils and monocytes (*R* ^2^: 0.61, 95% CI: 0.11–0.86, *p* = 0.02, Figure 2D). Additionally, analysis of DEGs in the gene signatures of each myeloid lineage cell revealed that most of the DEGs were upregulated in the CLP group compared with the sham group (Figure 3).

### 3.3 Dendritic Cells, Monocytes and Neutrophils Were Observed in Hippocampus of CLP Mice

**FIGURE 4:**
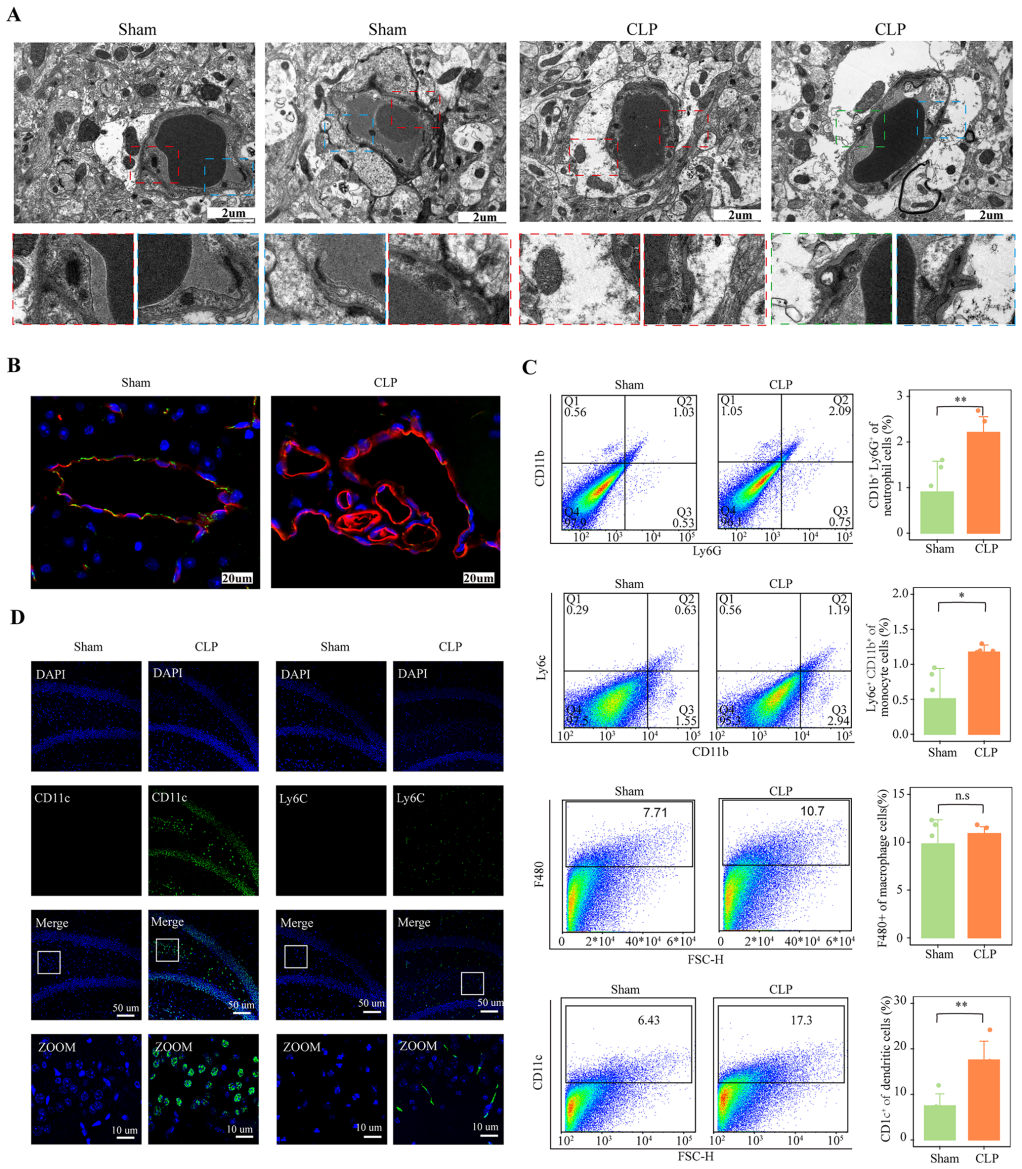
Blood–brain barrier damage and infiltration of dendritic cells, monocytes and neutrophils in the hippocampus of CLP mice. (A) Electron microscopy showing endothelial separation (red box), damaged tight junctions (blue box) and autophagosomes (green box) in CLP mice. (B) Immunofluorescence indicated the lower level of ZO‐1 (green) in CLP. (C) Flow cytometry revealed significantly increased proportions of neutrophils, monocytes and dendritic cells in CLP mice (mean ± SD, *p < 0.05, **p < 0.01, ^n.s^ *p* > 0.05). (D) Immunofluorescence confirmed higher proportions of monocytes and dendritic cells in CLP mice. CLP, cecal ligation and puncture.

Previous studies have shown myeloid cell infiltration in the hippocampus of mice after CLP, with a positive correlation between pDCs and aDCs, as well as neutrophils and monocytes. To verify the immune cell infiltration in the brains of CLP mice, the damage to the BBB was first assessed using electron microscopy. The endothelium and basal layer of brain vessels in CLP mice were separated, revealing noticeable edema in the interstitial tissue around the vessels. The tight junctions of the endothelial cells were swollen and damaged, and a small number of autophagosomes were observed in the tissues. The red box indicates the gap between the endothelium and the basal layer, the blue box highlights the tight junction and the green box shows the autophagosome (Figure 4A, *n* = 3). In addition, the levels of occluding and zonula occludens‐1 (ZO‐1) (green) were lower in CLP mice compared to the Sham one (Figure 4B, *n* = 3).

Flow cytometry analysis of hippocampal tissue revealed a significant increase in the proportion of neutrophils, monocytes and dendritic cells in the CLP group compared to the control group (*n* = 3). Although no significant difference was observed in macrophage populations, there was a trend toward an increase in the CLP group (Figure 4C, *n* = 3). Further immunofluorescence staining for CD11c^+^ dendritic cells and Ly6C^+^ monocytes confirmed that CLP mice had higher numbers of these immune cells in the hippocampus, whereas the sham group showed fewer (Figure 4D, *n* = 3).

### 3.4 Immune‐Related Hub Genes Were Positively Associated With Myeloid Cells Infiltration in Hippocampus of CLP Mice

**FIGURE 5:**
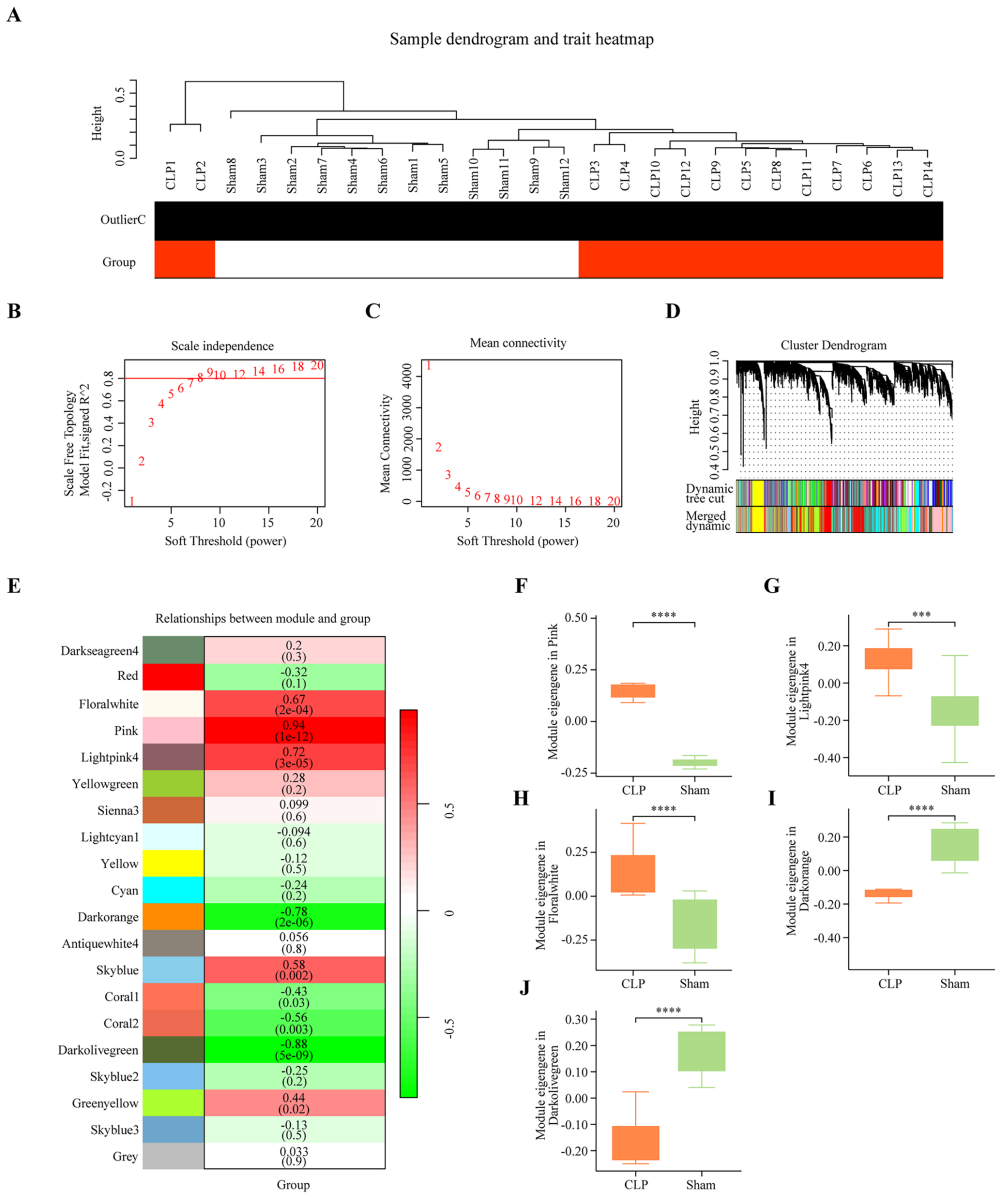
Identification of five modules significantly associated with CLP. (A) No samples were removed after flashClust analysis. (B, C) Scale independence and mean connectivity for all samples (*β* = 9). (D) Gene dendrogram and merged modules. (E) Heatmap showing correlations between individual modules and CLP. (F–J) Differences in module eigengenes between the sham and CLP groups for selected modules (mean ± SD, ***p < 0.001. ****p < 0.0001). CLP, cecal ligation and puncture.

**FIGURE 6:**
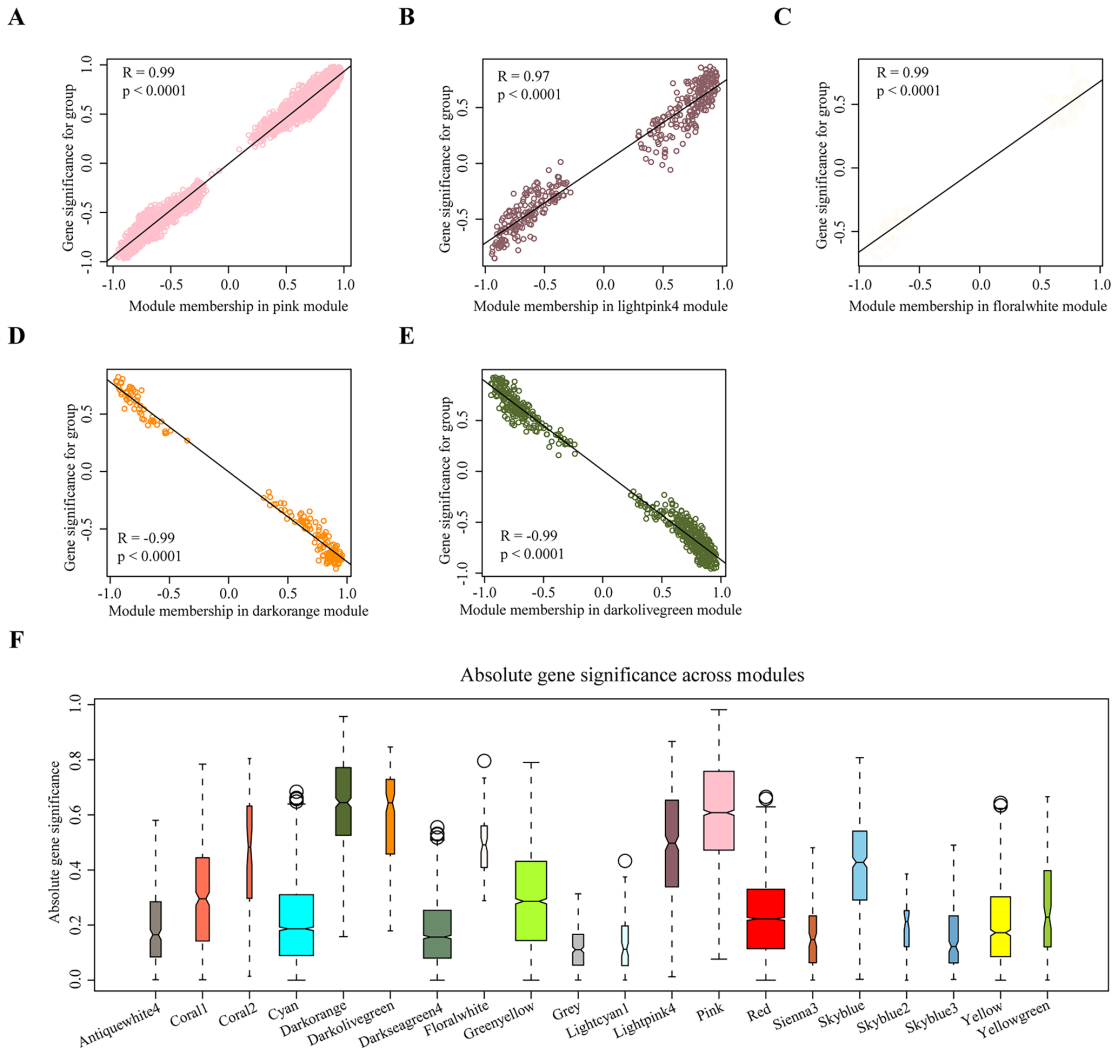
Gene significance and module membership in selected modules associated with CLP. (A–E) Correlation between module membership and gene significance for five selected modules. (F) Boxplot showing trait‐based module significance (average gene significance) across all groups and modules. CLP, cecal ligation and puncture.

**FIGURE 7:**
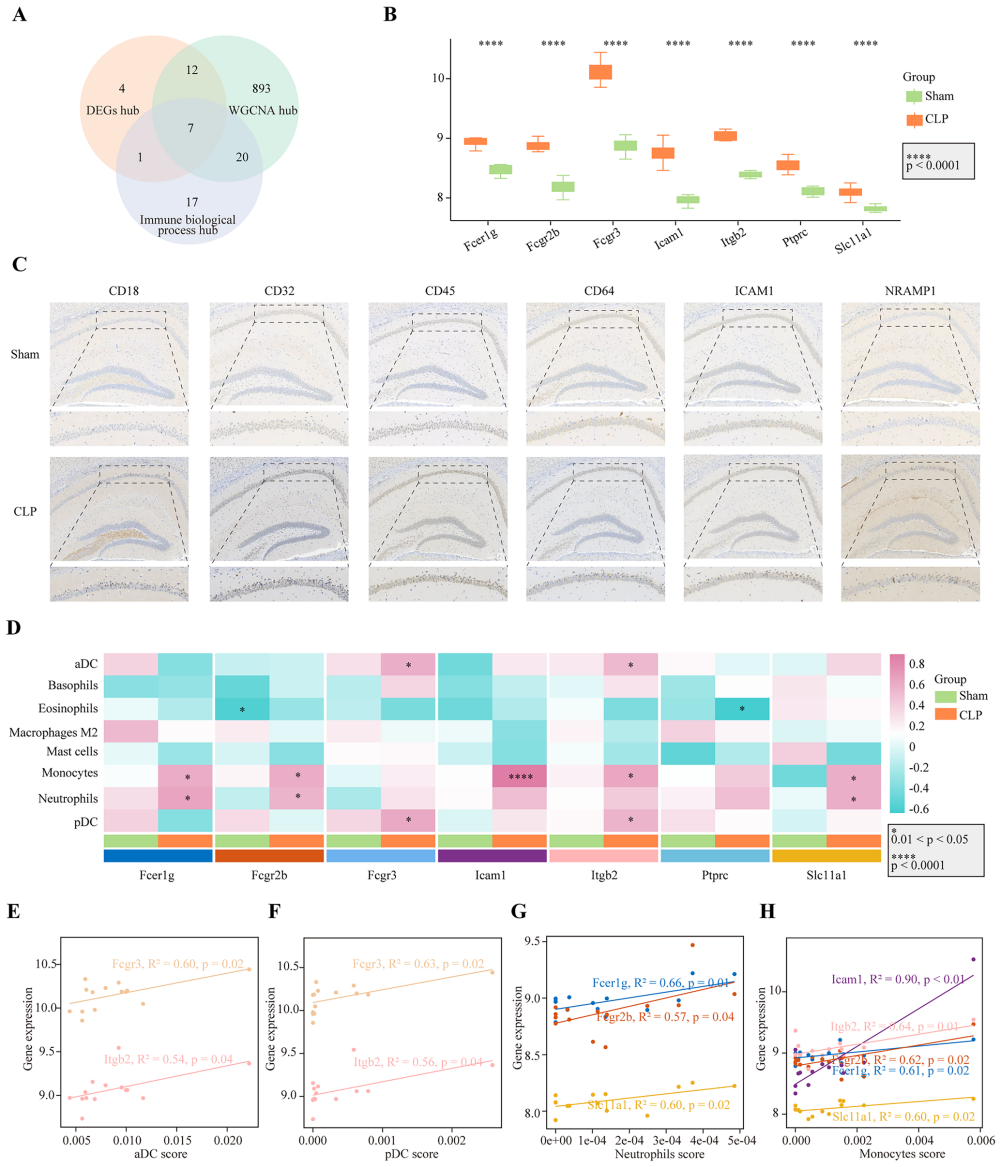
Hub gene selection and correlation with myeloid immune cell infiltration scores. (A) Identification of seven hub genes. (B) Boxplot showing the expression of hub genes in the sham and CLP groups (median ± IQR, ****p < 0.0001). (C) Protein expression of hub genes was elevated in CLP tissues. (D–H) Correlations between hub genes and myeloid immune cell infiltration scores. Fcgr3 and Itgb2 were positively correlated with aDCs and pDCs. Fcer1g, Fcgr2b and Slc11a1 were positively correlated with monocytes and neutrophils. Icam1 and Itgb2 were positively correlated with monocytes (*p < 0.05, ****p < 0.0001). aDCs, activated dendritic cells; CLP, cecal ligation and puncture; pDCs, plasmacytoid dendritic cells.

Seven immune‐related hub genes (*Fcer1g*, *Fcgr2b*, *Fcgr3*, *Icam1*, *Itgb2*, *Ptprc* and *Slc11a1*) were identified through an integrated approach involving three methods. From the PPI network, 24 hub genes were screened. In addition, 205 immune‐related biological processes were identified in which DEGs were enriched. Notably, 45 DEGs were enriched in more than 20 of these processes (Table 1). For further analysis, transcriptomic data from 16,629 genes were subjected to conjunction with WGCNA analysis. The standardised connectivity values for all 26 samples exceeded −5, and thus, all were included in the construction of the gene expression network (Figure 5A–C). This process resulted in the identification of 20 gene expression modules, which were merged based on a correlation coefficient greater than 0.75 (Figure 5D). Five of these modules were significantly correlated with the CLP group: pink (*R* ^2^ = 0.94, *p* < 0.0001), lightpink4 (*R* ^2^ = 0.72, *p* < 0.0001), floralwhite (*R* ^2^ = 0.67, *p* < 0.0001) were positively correlated, whereas darkorange (*R* ^2^ = −0.78, *p* < 0.0001) and darkolovegreen (*R* ^2^ = −0.88, *p* < 0.0001) were negatively correlated (Figure 5E). Significant differences in GS were observed across all five modules between the sham and CLP groups (Figure 5F–J). Moreover, positive correlations between GS and MM were identified in pink (*R* ^2^ = 0.99, *p* < 0.0001), lightpink4 (*R* ^2^ = 0.97, *p* < 0.0001) and floralwhite (*R* ^2^ = 0.99, *p* < 0.0001) modules, whereas negative correlations were noted in darkorange (*R* ^2^ = −0.99, *p* < 0.0001) and darkolovegreen (*R* ^2^ = −0.99, *p* < 0.0001) modules. The absolute GS value of the modules was presented in Figure 6F. A total of 932 hub genes were identified. The integration of the three methods led to the selection of seven immune‐related hub genes (*Fcer1g*, *Fcgr2b*, *Fcgr3*, *Icam1*, *Itgb2*, *Ptprc* and *Slc11a1*), all of which were upregulated in the CLP group (Figure 7A,B). The corresponding proteins expressed by these genes are highly expressed in CLP mice (Figure 7C).

**TABLE 1:** Number of times the gene was screened by 12 algorithms in cytoHubba.

Genes	Times	Genes	Times	Genes	Times	Genes	Times	Genes	Times
Itgb2	10	Cxcl1	5	P2ry6	2	Fam64a	1	Nrk	1
Ptprc	10	Il2rg	5	Prg4	2	Fam83d	1	P2ry12	1
Tnf	10	Itgal	5	Serpinb1a	2	Fbln5	1	Pbk	1
Cd14	9	Spi1	5	Serpinb1b	2	Fpr2	1	Pilrb2	1
Cybb	9	Csf3	4	Serpine1	2	Galnt15	1	Pla2g3	1
Fcer1g	9	Dpp4	4	Timp1	2	Gcnt4	1	Rin3	1
C1qa	8	Ms4a6d	4	Vwf	2	Gipr	1	Serpina3f	1
Fcgr3	8	Plaur	4	A2m	1	Glp2r	1	Serpina3g	1
Hck	8	Spint1	4	Acaa1b	1	Gm694	1	Serpina3i	1
Icam1	8	Agt	3	Ada	1	Gpr65	1	Serpina3n	1
Mmp9	8	C3ar1	3	Alox5	1	Gpx3	1	Serpind1	1
Sell	8	Elf5	3	Alox5ap	1	Hsf3	1	Sgk1	1
Ccl3	7	Glycam1	3	Angptl4	1	Igj	1	Sgk3	1
Ctss	7	Itga2	3	Batf	1	Il33	1	Sla	1
Fcgr4	7	Lao1	3	C5ar1	1	Inmt	1	Slc22a21	1
Slc11a1	7	Plau	3	Cd209a	1	Itgad	1	Slc25a34	1
Tlr7	7	Plet1	3	Cd72	1	Lrrc25	1	Slc40a1	1
C1qb	6	Siglec1	3	Clec4a2	1	Ly6c1	1	Socs3	1
Ccl4	6	AF251705	2	Cnr2	1	Ly6c2	1	Spn	1
Fcgr2b	6	Bcl2a1b	2	Col6a4	1	Mmp8	1	St14	1
Igsf6	6	Cd52	2	Creb5	1	Ms4a6b	1	Tcap	1
Lyz2	6	Clec4a3	2	Csf2rb	1	Ms4a6c	1	Tlr12	1
Pirb	6	Csf2rb2	2	Cxcr2	1	Msr1	1	Tnc	1
Rac2	6	Cyp4a10	2	Depdc1b	1	Muc1	1	Tnfsf8	1
C1qc	5	Lox	2	Dok2	1	Myo1g	1	Trem1	1
Ccr2	5	Ly6a	2	Dpep2	1	Ngp	1	Ucp2	1

Next, we investigated the association between the seven hub genes and the immune infiltration scores of eight different myeloid lineage cell types. The results are presented in Figure 7C. In the CLP group, *Fcgr3* and *Itgb2* were positively correlated with aDCs and pDCs (Figure 7D–F). These findings were consistent with the observed positive correlation between the immune infiltration scores of aDCs and pDCs, suggesting that *Fcgr3* and *Itgb2* may play a role in mediating this relationship. Similarly, *Fcer1g*, *Fcgr2b* and *Slc11a1* were positively correlated with monocytes and neutrophils (Figure 7D,G,H), further supporting the observed positive correlation between the immune infiltration scores of these cell types. Additionally, *Icam1* and *Itgb2* were found to be positively correlated with monocytes (Figure 7H).

## 4 Discussion

The pathophysiology of midbrain inflammation in SAE patients is complex. A deeper understanding of the interplay between systemic diseases and brain inflammation could facilitate the development of effective therapeutic strategies for SAE management. One key contributor to the overactivation of intracranial inflammation and subsequent neuronal damage is the infiltration of peripheral immune cells into the brain, which plays a critical role in the pathogenesis of SAE. In this study, we observed significant differences in the infiltration of myeloid immune cells in the hippocampus of septic mice. Furthermore, we identified strong positive correlations between activated aDCs and pDCs, as well as between monocytes and neutrophils. Seven immune‐related hub genes were identified, of which *Fcgr3* and *Itgb2* were positively correlated with aDCs and pDCs, while *Fcer1g*, *Fcgr2b* and *Slc11a1* were positively correlated with monocytes and neutrophils. These results suggest that these genes may play pivotal roles in the coordinated immune responses among these cell types. Collectively, our findings enhance the understanding of peripheral immune cell infiltration in the brain and propose potential therapeutic targets for SAE.

Uncontrolled neuroinflammation can lead to the dysfunction and apoptosis of various brain cell types, including microglia, neurons and endothelial cells, thereby significantly contributing to the pathogenesis of SAE [28]. The activation of resident brain immune cells triggers both peripheral and local inflammatory responses, exacerbating neuroinflammation and worsening sepsis outcomes [9, 29, 30]. In our analysis, we observed significant infiltration of myeloid immune cells, particularly dendritic cells, neutrophils and monocytes, in the hippocampus of mice after cecal ligation and puncture. Disruption of the BBB is a well‐established route for the entry of inflammatory cells into the brain [9], with early BBB compromise observed in rodent models of sepsis [31]. A previous study highlighted that sepsis is associated with an increase in leukocyte rolling and adhesion in the brain microcirculation, which subsequently leads to the accumulation of neutrophils in the brain [9]. Neutrophils enter the brain following initial binding to endothelial cells via selectins, followed by neutrophil activation through the chemokine receptor CXCR2, resulting in integrin activation and firm adhesion [9]. The subsequent transmigration of neutrophils across the endothelial barrier leads to vascular barrier disruption [32]. In addition to neutrophils, other immune cells, such as inflammatory monocytes, are recruited, although their precise roles in this process remain unclear [33]. Inflammatory monocytes utilise integrins for recruitment, with the key distinction being their activation through the chemokine receptor CCR2, followed by adhesion to the vascular wall [34]. Studies have shown that CCR2 is a critical cytokine involved in monocyte/macrophage chemotaxis [35, 36]. For instance, intermedin inhibits the chemotaxis of monocytes/macrophages to peripheral organs by reducing CCR2 expression, thereby preventing inflammatory cell infiltration into peripheral tissues and mitigating acute organ damage caused by the inflammatory response [36]. In our study, *Cxcr2* and *Ccr2* were significantly upregulated in the hippocampus of CLP mice, suggesting that these molecules may represent potential targets for inhibiting immune infiltration in the brain. Furthermore, this study provides novel evidence of dendritic cell infiltration in the brain following CLP, a finding that has rarely been reported in previous studies. This adds an additional layer of complexity to the inflammatory response, warranting further investigation into the mechanisms underlying dendritic cell involvement in neuroinflammation.

In this study, seven immune‐related hub genes (*Fcer1g*, *Fcgr2b*, *Fcgr3*, *Icam1*, *Itgb2*, *Ptprc* and *Slc11a1*) were identified. *Fcer1g*, *Fcgr2b* and *Fcgr3* encode Fc receptors for Ig, which are immunoglobulin superfamily membrane proteins expressed by a wide range of innate and adaptive immune cells. *Fcer1g* encodes the Fc γ receptors, which form non‐covalent complexes with several immunoglobulin Fc region‐binding receptor complexes, including FcγRI, FcγRIIB (encoded by FcγR2b), FcγRIII (encoded by Fcgr3) and FcγRIV [37, 38, 39, 40]. Innate immune effector cells express both activating and inhibitory FcγRs. In mice, monocytes and macrophages exhibit broad expression patterns encompassing all FcγRs (FcγRI‐IV), while dendritic cells predominantly express FcγRI, FcγRIIB and FcγRIII. FcγRs act as a pivotal role in modulating various immune responses, and may therefore represent promising targets for innovative immunotherapeutic strategies [41]. These receptors are crucial in facilitating leukocyte recruitment and activation, bridging the antibodies generated by the adaptive immune system with the effector functions of the innate immune system [41]. Notably, FcγRIIB is responsible for inhibiting the signal cascade induced by activating FcγRs [41] and reduced or absent expression of FcγRIIB has been linked to the development or exacerbation of autoimmune diseases [42]. In our analysis, *Fcer1g*, *Fcgr2b* and *Fcgr3* were all upregulated after CLP surgery. *Fcer1g* and *Fcgr2b* were positively correlated with neutrophils and monocytes, suggesting their significant role in the activation of these cell types. *Fcgr3* was positively correlated with dendritic cells, indicating its involvement in dendritic cell activation. *Icam1*, an adhesion molecule found on endothelial cells, interacts with integrins expressed by immune cells and plays a critical role in several cell‐contact‐dependent processes, including leukocyte‐endothelial adhesion and antigen presentation [43]. Our results revealed a significant correlation between the expression of *Icam1* and the increase in monocytes in the hippocampus of CLP mice, suggesting that *Icam1* may play a critical role in monocyte extravasation. *Itgb2* encodes a β2 integrin subunit, also known as CD18, which is specifically expressed by leukocytes and plays an important role in leukocyte recruitment, migration and cellular interactions [44, 45, 46]. Furthermore, CD18 is strongly implicated in immune system regulation and can have negative or positive effects depending on its activation state [47]. *Slc11a1*, a divalent cation transporter expressed in monocyte lineage cells, is crucial for the innate resistance of mice against infections by intracellular microbes [48, 49]. *Slc11a1* ^+/+^ monocytes have been shown to contribute to the recruitment of inflammatory cells to the brain during *Salmonella* infection [50]. In this study, the positive correlation between *Slc11a1* and neutrophils and monocytes suggests that it may play a role in the recruitment of these cells to the site of inflammation.

However, this study has several limitations. First, while we analysed immune cell infiltration in the hippocampal tissue of mice 24 h post‐CLP using transcriptomic methods, we did not assess the temporal dynamics of immune cell infiltration across different stages of sepsis. The immune response may vary over time, and a longitudinal analysis could provide valuable insights into the progression of brain inflammation in sepsis. Second, our observations were limited to immune cell infiltration in the hippocampus; we did not extend our examination to other regions of the brain. Different brain areas may exhibit distinct immune responses, which could influence the overall pathophysiology of SAE. Third, while we identified immune cell infiltration patterns, the underlying mechanisms driving this infiltration remain unclear. Further research is needed to elucidate the molecular and cellular mechanisms that regulate peripheral immune cell trafficking to the brain during sepsis. Future studies will focus on understanding the role of peripheral immune cell infiltration in the pathogenesis of SAE.

## 5 Conclusion

The results of this study demonstrated that myeloid lineage cells, including monocytes, neutrophils and dendritic cells, infiltrate the central nervous system during sepsis. This highlights the critical role of peripheral immune cell infiltration in the pathogenesis of SAE and underscores the potential contribution of these cell types to brain inflammation. The hub genes identified in this study may serve as promising targets for future research and therapeutic interventions aimed at modulating immune responses in SAE. These insights enhance our understanding of the complex interplay between systemic infection and brain inflammation, offering hope for improved outcomes in patients with SAE.

## Author Contributions

**Hui Zhang:** conceptualization (equal), formal analysis (lead), methodology (lead), visualization (lead), writing – original draft (lead), writing – review and editing (lead). **Meixian Zhang:** data curation (supporting), formal analysis (supporting). **Fanbing Meng:** conceptualization (supporting), data curation (supporting), formal analysis (supporting). **Xiaoxiao Sun:** conceptualization (supporting), formal analysis (supporting). **Miaomiao Fei:** conceptualization (equal), supervision (equal), writing – review and editing (equal). **Lize Xiong:** conceptualization (lead), supervision (lead), writing – review and editing (lead). **Cheng Li:** conceptualization (lead), investigation (lead), resources (lead), writing – review and editing (lead). **Yanan Gao:** writing – review and editing (equal). **Qian Chen:** writing – review and editing (equal). **Qiang Liu:** writing – review and editing (equal).

## Ethics Statement

All mice treatments obeyed the rules of the Ethics Committee of Shanghai Fourth People's Hospital, affiliated with Tongji University (ethical committee number: TJBH07024104).

## Conflicts of Interest

The authors declare no conflicts of interest.

## Supporting information


**Figure S1:** Saturation curves for read counts and detected genes.

**Figure S2:** An overview of all samples. (A). Correlation heatmap across all samples. (B). Boxplot of normalised gene expression across all samples.

**Figure S3:** KEGG enrichment analysis of DEGs.

## Data Availability

The raw sequence data generated in this study have been deposited in the Genome Sequence Archive (GSA: CRA016410) at the National Genomics Data Center, China National Center for Bioinformation/Beijing Institute of Genomics, Chinese Academy of Sciences. These data are publicly accessible at https://ngdc.cncb.ac.cn/gsa.
