# Supplementary figures and images for: Peripheral Immune Cell Infiltration in the Hippocampus of Sepsis Mice
Source: J Cell Mol Med. 2025 Oct 31;29(21):e70872. doi: 10.1111/jcmm.70872 (PMC12808816; doi:10.1111/jcmm.70872)

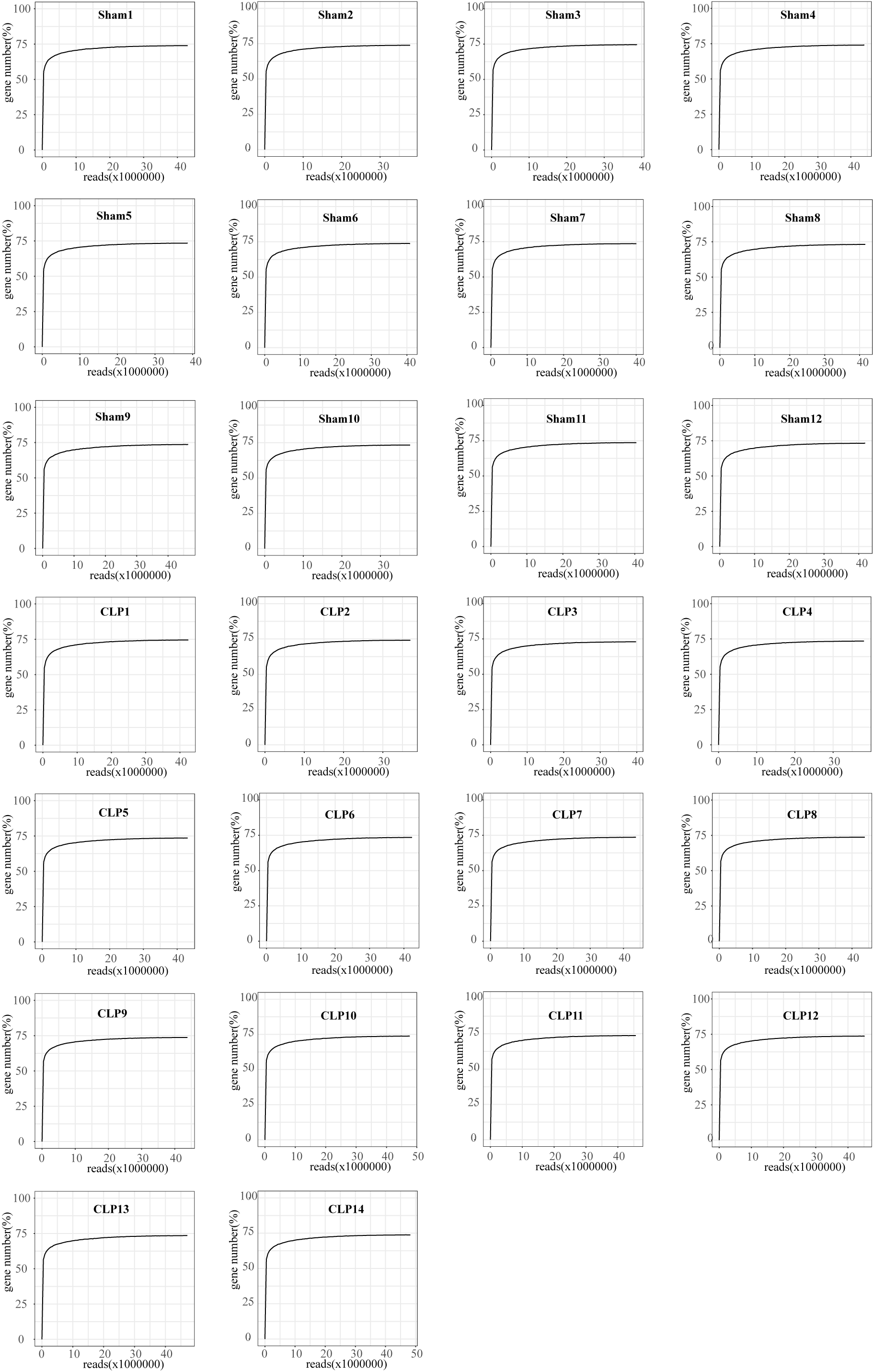

Supplement: Supplementary file 1 — Figure S1: Saturation curves for read counts and detected genes. [file JCMM-29-e70872-s003.tif]

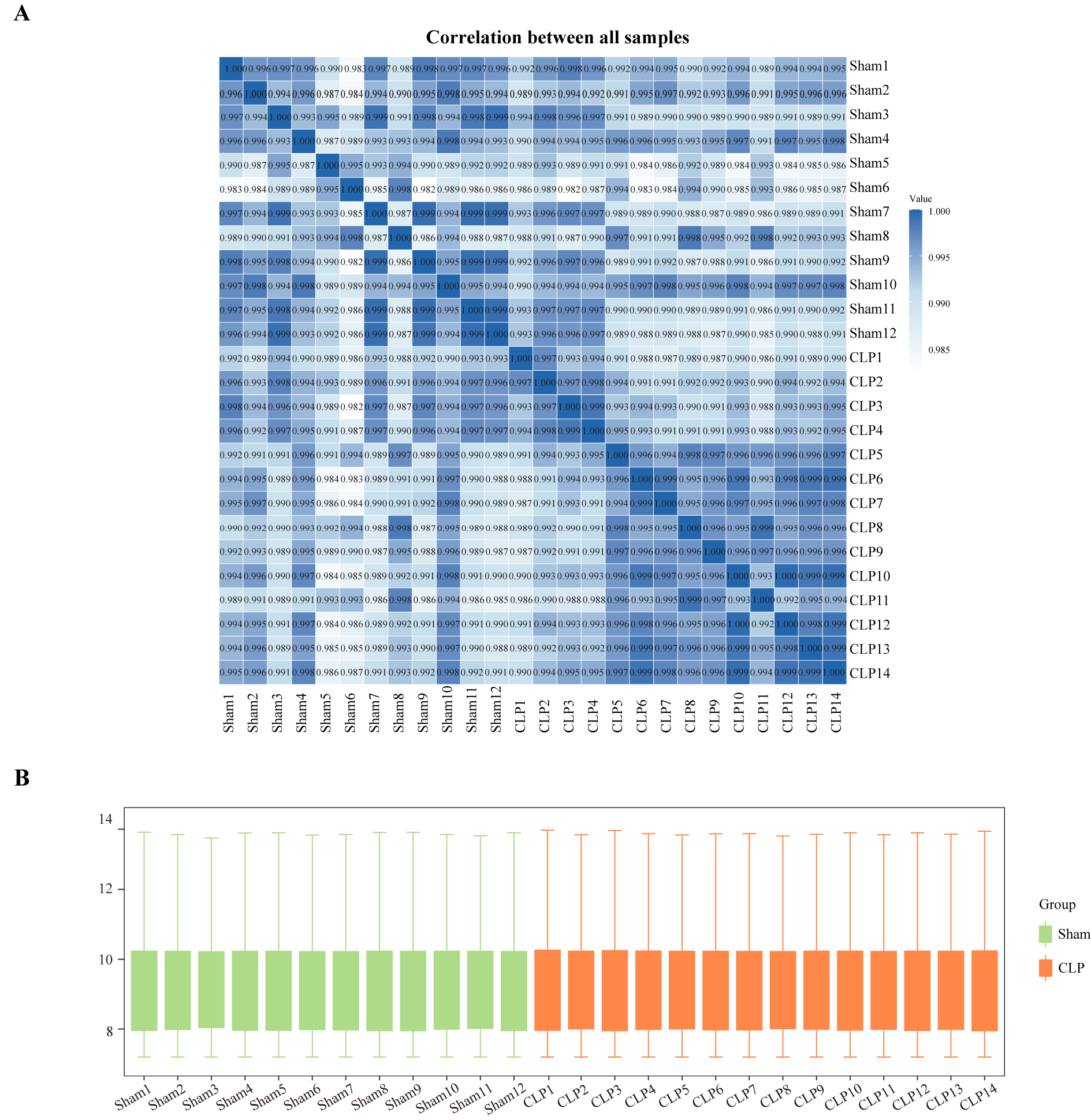

Supplement: Supplementary file 2 — Figure S2: An overview of all samples. (A). Correlation heatmap across all samples. (B). Boxplot of normalised gene expression across all samples. [file JCMM-29-e70872-s002.tif]

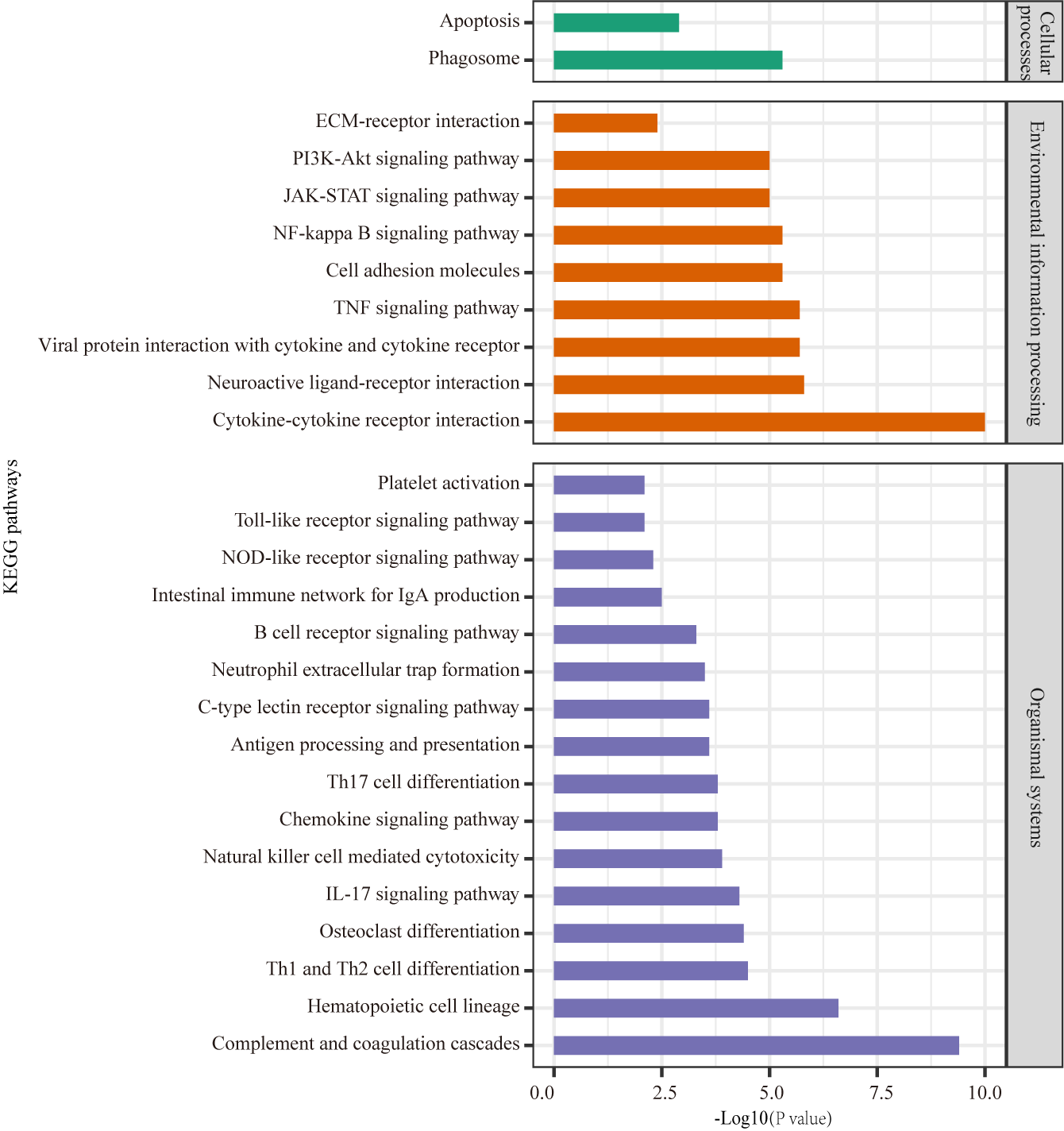

Supplement: Supplementary file 3 — Figure S3: KEGG enrichment analysis of DEGs. [file JCMM-29-e70872-s001.tif]
